# Psychosocial and behavioural interventions towards HIV risk reduction for serodiscordant couples in Africa: A systematic review

**DOI:** 10.4102/sajpsychiatry.v24i0.1136

**Published:** 2018-06-05

**Authors:** Sibongile Mashaphu, Jonathan K. Burns, Gail E. Wyatt, Naseema B. Vawda

**Affiliations:** 1 Department of Psychiatry, University of KwaZulu-Natal, South Africa; 2 University of Exeter, United Kingdom; 3 Department of Psychiatry and Bio-behavioural Sciences, University of California Los Angeles, United States; 4 Department of Behavioural Medicine, University of KwaZulu-Natal, South Africa

## Abstract

**Background:**

Sexual transmission of HIV frequently occurs in the context of a primary relationship between two partners; however, HIV prevention interventions generally focus on individuals at risk, rather than specifying couples as a unit of change and analysis, neglecting the crucial role that partners may play in sexual behaviour. This article reviews published scientific literature addressing couple-oriented HIV counselling and testing and other behavioural interventions using an online search for peer-reviewed papers.

**Methods:**

A systematic review was conducted to evaluate what has been published on psychosocial interventions in HIV serodiscordant couples in Africa. Electronic databases were searched from January 1990 to December 2015. Quality assessment of included studies was conducted using the Systematic Appraisal of Quality in Observational Research tool.

**Results:**

The electronic database searches initially retrieved 493 records; after cross-referencing, removing duplicates and applying strict inclusion and exclusion criteria, only eight papers were included in this review. All the studies under review showed that couples-focused counselling and educational programmes were associated with positive outcomes including reduced HIV transmission, reduced unprotected sex, increased rates of status disclosure and high levels of treatment adherence.

**Conclusions:**

The literature on interventions for HIV serodiscordant couples is sparse. However, most interventions indicate that couples-focused interventions are effective in HIV risk reduction. In spite of the limited available data and repeated recommendations by different health authorities, couple-centred approaches to HIV prevention have not been implemented on a large scale.

## Introduction

In 2014, the Joint United Nations Programme on HIV/AIDS (UNAIDS) announced bold targets for the global response to the human immunodeficiency virus (HIV), known as the 90–90–90 strategy, that aims to reduce the disease to a low-level endemic disease by 2013.^1,2,3^ However, moving the global response towards this target will pose huge challenges to public health systems in resource-limited settings such as South Africa and other low-income countries. This calls for innovative, cost-effective and culturally appropriate behavioural approaches to augment the current biomedical treatments. The argument that HIV is a social and behavioural problem gives insight into many possible ways in which mental health care workers can contribute positively towards the described global targets.^4^

HIV and AIDS remain major contributors to the high burden of communicable diseases in Africa.^5^ However, with the advent of antiretroviral (ARV) treatment, HIV and AIDS are now a relatively more treatable chronic illness with more HIV-positive people seeking to have a good quality of life. This implies that the prevalence of serodiscordant couples, or mixed HIV status couples where one partner is HIV positive and the other one is not, may increase with time. The current literature indicates that the prevalence of HIV discordance among married and co-habiting couples in Africa is high, ranging from 3% to 20% in the general population to 20% – 35% within couples in which one partner seeks HIV care services.^6^

There is a growing consensus that HIV prevention research should address couples as a unit of behaviour change and intervention.^7^ In sub-Saharan Africa, HIV-negative members of discordant couples are at extremely high risk of HIV infection with 30% of all new infections occurring in this group of individuals.^8^

However, given the high proportion of incident HIV infections in sub-Saharan Africa which occur within married HIV serodiscordant couples, very few interventions currently target couples as a unit of change.^9^ Interpersonal dynamics between partners tend to be overlooked in HIV prevention models.^10^ Examining the broader literature on partner influences in health behaviour demonstrates that partners and accompanying relationship factors need to be included in how we conceptualise health behaviour change.^10,11^

Research on the experiences of both heterosexual and homosexual HIV serodiscordant couples in the United States has highlighted their unique challenges regarding sexual intimacy, disclosure to family and friends, feelings of isolation, uncertainty about the future, trust, commitment and reproductive decisions.^12^ Relationship stress increases dramatically when one partner becomes positive and the other is not, with many previously stable relationships becoming abusive after HIV status disclosure.^13^ Sources of conflict are generally centred on how the illness was acquired, and concerns about transmission and guilt.^14^ Negotiating safer sex practices was also reported to be greatly affected by cultural norms and gender power dynamics.^15,16^ A study on serodiscordant couples in Uganda highlighted the need to address gender power imbalances when counselling couples. This study reported that the effectiveness of any programme promoting condom use depends on its acceptance by the male partner because some men interpreted the request to use a condom as insulting, mistrustful and a barrier to sexual fulfilment.^17^ As a consequence of these many challenges, psychological distress, such as depression, anxiety and high suicide rates, has been reported in serodiscordant couples.^18^

A study that examined the knowledge and challenges of living with HIV serodiscordance in Uganda revealed that most participants lacked accurate knowledge about discordance.^9^ Their lack of clarity on HIV discordance rendered them highly susceptible to popular myths and misconceptions.^9^

There is substantial evidence in the literature that voluntary HIV counselling and testing can affect sexual behaviours, and a multi-country trial suggested that voluntary counselling and testing (VCT) is cost-effective and efficacious in promoting behaviour change, particularly in high HIV prevalence settings.^19^ A few studies have indicated that couples voluntary counselling and testing (CVCT) significantly decreases HIV transmission within couples.^20^ Given the above, it is imperative that HIV prevention and intervention efforts also focus on couples as units of change and analysis, emphasising the role that partners may play in HIV transmission instead of focusing prevention efforts only on individuals at risk. In this study, we sought to review all the literature that has been published on serodiscordant couple intervention over the last quarter of this century. The aim was to appraise and synthesise all the empirical evidence that exists on the different interventions that have been used for serodiscordant couples, specifically in Africa, the continent that carries the highest burden of HIV infections. We also aimed to provide a narrative summary and a critical analysis of the studies that were included. The long-term aim of the research team is to design a culturally congruent behavioural intervention to address the unique psychological, physical and other health needs of HIV serodiscordant heterosexual couples in South Africa.

## Methods

### Search strategies

The following electronic databases were searched by two reviewers in 2016: PubMed/Medline, EBSCOhost, AJOL (African JournalsOnline) and SABINET. The search string used was as follows: (HIV OR AIDS) AND (mixed status OR serodiscord*) AND (couple OR partner OR relationship OR married OR cohabiting) AND (intervention OR counsel* OR therapy OR psychotherapy OR risk reduction) AND (Africa). This search string was used for PubMed and SABINET with modifications where necessitated or restricted by specific database options. The other databases used terms specific to HIV and AIDS serodiscordance and the intervention focus, ‘couple, relationship, married, cohabiting’. The electronic databases were searched for titles or abstracts containing these terms in all published articles between January 1990 and December 2015 inclusive. Only studies published in English were included. The reference lists of all included studies were hand-searched for additional relevant reports which were added until no further publications were found.

### Inclusion and exclusion criteria

As the focus of this review was on research conducted in Africa, only papers reporting data from African countries were included. All studies meeting all of the following criteria were included: (1) those including a population of couples with serodiscordant HIV status; (2) those reporting on an intervention; and (3) those that had sufficient information available in English to interpret the study. Exclusion criteria were as follows: (1) studies not including couples with serodiscordant HIV status; (2) those not including an intervention; and (3) unpublished data of any form including conference proceedings, case reports, dissertations and publications reporting duplicate data from the same population.

### Data extraction and analysis

Two reviewers (S.M. and J.K.B.) independently extracted data from selected studies that met all inclusion criteria; data were extracted onto a customised data-extraction sheet that included sample characteristics and study design, the primary aim of the intervention, the content of the intervention and the main outcomes of the intervention. Data were described and analysed according to broad themes related to the nature of the intervention and outcomes being assessed.

### Quality assessment of included studies

Quality assessments were performed independently by two investigators (S.M. and J.K.B.) using the Systematic Appraisal of Quality in Observational Research (SAQOR) tool that comprises six domains (each containing two to five questions): sample, control or comparison group, exposure or outcome measurements, follow-up, confounders and reporting of data (Table 1).^21^ Then, in a consensus meeting of the two investigators, a summary quality assessment was assigned for each of the six domains, and then an overall summary grade was determined based on adequacy in these domains. The overall quality of the study was graded as high, moderate, low or very low.

**TABLE 1:** Quality assessment of included studies using the Systematic Appraisal of Quality in Observational Research tool.

References	Quality of sample	Control or comparison group	Quality of exposure/outcome	Follow-up	Distorting influences	Reporting of data	Summary quality rating of study
Haberer et al.^22^	Adequate	Adequate	Adequate	Unclear	Adequate	Adequate	High
Coates et al.^23^	Adequate	Adequate	Adequate	Adequate	Adequate	Adequate	High
Montgomery et al.^24^	Adequate	Unclear	Unclear	N/A	N/A	Unclear	Low
Allen et al.^17^	Unclear	Unclear	Adequate	N/A	Unclear	Adequate	Moderate
Kairania et al.^25^	Adequate	N/A	Adequate	N/A	Inadequate	Unclear	Moderate or low
Allen et al.^16^	Adequate	N/A	Adequate	Adequate	Unclear	Unclear	Moderate
Jones et al.^26^	Adequate	Adequate	Adequate	Unclear	Adequate	Unclear	High
Allen et al.^27^	Unclear	Unclear	Adequate	Unclear	Adequate	Unclear	Moderate

N/A, not applicable.

## Etchical consideration

Ethical approval was obtained from the University of KwaZulu-Natal’s Biomedical Research Ethics Committee (BREC # BFC 166/15).

## Results

**FIGURE 1:**
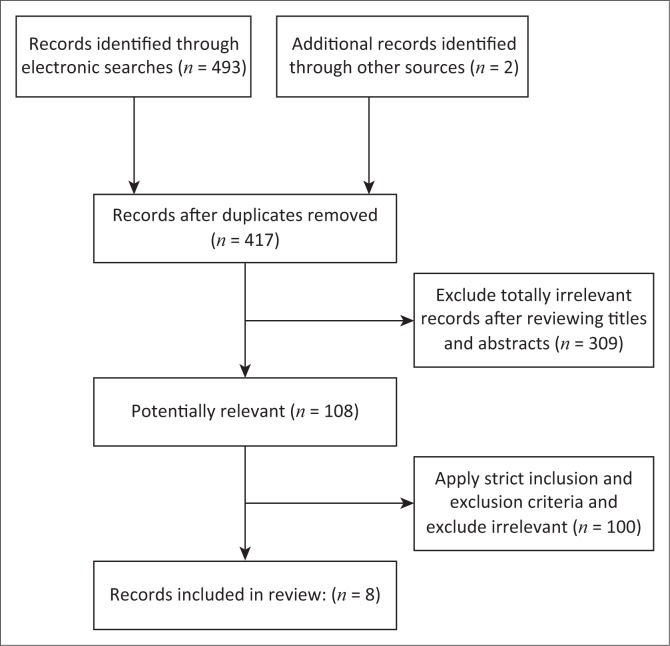
Flow diagram for systematic review process.

The electronic database searches initially retrieved 493 records (395 from PubMed, 3 from AJOL, 58 from Sabinet and 37 from EBSCOhost). Cross-referencing retrieved an additional 2 records from primary studies which were cross referenced for further citations, leaving a total of 495 papers to review. After the removal of 76 duplicates and the exclusion of 309 totally irrelevant papers, 108 potentially relevant papers were left and full text obtained. With the strict application of inclusion and exclusion criteria, only eight papers met criteria for inclusion in this review (Figure 1).

### Description of included studies

Eight studies published between 1990 and 2015 reporting interventions in HIV serodiscordant couples were included in this review (see Table 2 for characteristics of included studies). All studies took place in Africa. The papers were grouped into four categories or themes based on the nature of the intervention and outcomes being assessed.

**TABLE 2:** Couple-centred psychosocial and behavioural interventions in HIV serodiscordant couples in Africa from the literature published between 1995 and 2015.

Author	Location, year	Primary intervention aim	Content of the intervention	Study design	Sample size	Inclusion and exclusion criteria	Main findings
**Adherence to antiretroviral prophylaxis**							
Haberer, et al.^22^	Uganda (Kabwohe; Kampala; Toro) 2009	Investigate levels of adherence to antiretroviral prophylaxis and its correlation to HIV infection in serodiscordant couples	Couples received risk reduction counselling, couples counselling and condoms	Cohort study: Placebo controlled ‘convenience sample’	*N* = 1147 individual HIV-negative partners in Uganda	Inclusion: Heterosexual serodiscordant couples attending clinical research sites in Kenya and Uganda	High levels of PrEP adherence, when combined with active adherence monitoring, counselling and support, were associated with a high level of protection from HIV acquisition by the HIV-negative partner (99.1% and 97.2% measured by unannounced pill counts and electronic monitoring, respectively). 0% HIV seroconversion for the active group 14 infections occurred in the placebo group
**VCT as a preventative strategy**							
Coates, et al.^23^	Nairobi, Kenya; Tanzania and Trinidad	Determine the efficacy of HIV VCT in reducing unprotected intercourse among individuals and sexual-partners	Individuals or couples VCT versus basic health information, watching videos, provision of condoms	Longitudinal study Random sampling	*n* = 3120 individuals *n* = 586 couples	Inclusion: ≥ 18 years old; planning to remain in catchment area for 1 year; not known to be infected with HIV-1 participants’ spouses = secondary participants	Individual men and woman who received VCT were significantly more likely to reduce unprotected intercourse with non-partners (13% men and 17% woman) when compared to those assigned with health information. Among couples where one or both were HIV positive, unprotected intercourse was reduced compared with both HIV-negative couples.
**Couple-focused HIV prevention and couples-based volunteer counselling and testing**							
Montgo-mery, et al.^24^	Uganda and Zambia	Provide an empirical basis for understanding the mechanisms through which couple-focused HIV prevention works	Dyadic intervention by recruiting and interviewing couples together. Interviews with MDP 301 staff	Comparative qualitative study	Couples: *n* = 10 Staff members: *n* = 23	Inclusion: Uganda: serodiscordant couples booked together. Zambia: serodiscordant woman recruited alone. All women required to be sexually active. MDP301 trail staff.	Spouses’ transformation of motivation is strong where couples are recruited and both partners stand to gain considerably. Among serodiscordant couples in Uganda, communal coping was evidenced through joint consent to participate, regular couple counselling, workshop attendance, sharing of HIV results and strong spousal support for adherence and retention.
Allen, et al.^17^	Rwanda and Zambia, 2003	To determine the efficacy of an intervention for the promotion of CVCT	To determine the efficacy of an intervention for the promotion of CVCT	Intervention study	9900 couples 61 network agents	Couples	Acceptability of CVCT: 14% if invited couples. Invitations delivered at home are a stronger predictor of attendance to CVCT than community interventions.
Kairania et al.^25^	Rakai, Uganda	Evaluate the efficacy of a stepwise strategy to promote disclosure of HIV-positive results among discordant couples	Facilitated couples counselling; sensitisation meetings	Cohort study and random trails	*N* = 337 9293 HIV-discordant couples; 22 concordant couples; 22 negative couples0	Inclusion: Couples	iThe facilitated couple counselling approach to disclosure resulted in high rates (80.9%) of disclosure, irrespective of the sex of the HIV-positive partner, dispelling past studies that suggest lower disclosure rates among HIV-positive woman than men. iA phased approach of sensitisation meetings; individual level counselling and open disclosure of results identified as key factors.
Allen et al.^16^	Lusaka, Zambia, August 1994–November 1998	Assess sexual behaviour of cohabitating heterosexual discordant couples following VCT	Sexual diary; interview of individuals and then couples; additional counselling if unprotected sex was reported; laboratory tests for syphilis and gonorrhoea	Comparative, longitudinal study comparing condom use before and after joint VCT	*N* = 963 cohabitating heterosexual discordant couples	Inclusion: Cohabitating in a sexual relationship for at least 6 months; residing in Lusaka; women ≤ 48 years and men ≤ 65 years of age	In discordant couples, at baseline less than 3% reported current condom use compared to > 80% reported condom use encounters after VCT. Condom use was reported in only 28% of sexual exposures within concordant couples. Despite self-reporting > 80% of reported acts of intercourse in discordant couples included condom use, 87% of new HIV infections were acquired from the participants’ spouse.
Jones et al.^26^	Lusaka, Zambia, May 2006–February 2010	Assess if an HIV risk-reduction intervention would influence sexual behaviour and decrease risk behaviour when delivered in a couple or individual format	Four intervention sessions lasting 2 h delivered weekly	Intervention study, longitudinal	*N* = 216 HIV seroconcordant and discordant couples. *n* = 108 couples in individual format *n* = 108 couples in group format	Inclusion: Currently in heterosexual relationship for 6 months or more; residing in Lusaka, Zambia urban district; 18 years or older; one member is HIV-seropositive	In both the group and individual situations, high levels of acceptability and willingness to use barriers predicted sexual barrier use. Positive communication and IPV decreased over time. In both the group and individual situations, high rates of condom use at the time of the study and in the long-term follow-up highlight the positive impact of exposure to interventions.
**Risk reduction**							
Allen et al.^28^	Rwanda, 1988–1990	To assess the impact of an education and CVCT intervention on condom use and seroconversion	Educational videos; discussion groups	Prospective cohort study	*N* = 53 HIV serodiscordant couples	Inclusion: co-habituating couples with discordant HIV serology results	Condom use increased from 4% to 57% of couples over 1 year of follow-up. Lower seroconversion rate among couples in intervention compared with estimated seroconversion rates in the general population.

VCT, voluntary counselling and testing; PrEP, pre-exposure prophylaxis; CVCT, couples-based voluntary counselling and testing; IPV, intimate partner violence; MDP, microbicides developmental programme.

#### Adherence to antiretroviral treatment

In a cohort study in Kampala and Tororo, Uganda, Haberer et al.^22^ sought to investigate the levels of adherence to ARV prophylaxis and its correlation to HIV infection in serodiscordant couples. Within a randomised placebo-controlled trial of oral tenofovir among HIV-negative members of serodiscordant couples, the research team collected objective measures of pre-exposure prophylaxis (PrEP) adherence using unannounced home-based pill counts and electronic pill bottle monitoring. Participants received individual and couples-based adherence counselling at PrEP initiation and throughout the study. Counselling was intensified if unannounced pill count adherence fell to <80%. A total of 1147 HIV-negative participants were enrolled. Fourteen HIV infections occurred among the participants assigned to the placebo group, and 0% seroconversion rates occurred in the active group.

#### Voluntary counselling and testing as a preventive strategy

In Nairobi, Kenya, Coates et al.^23^ examined the efficacy of VCT as a preventative strategy in reducing unprotected intercourse among sexual partners. A longitudinal random sampling method was employed. Individuals and couples received VCT, whereas the comparison group received basic health information, watched videos and were provided with condoms. A total of 3120 individuals and 586 couples were enrolled. Individual men and women who received VCT were significantly more likely to reduce unprotected intercourse when compared to those who were assigned with health information. Among the couples where one or both were HIV positive, unprotected intercourse was reduced compared with HIV-negative couples.

#### Couples-focused HIV prevention and couples-based volunteer counselling and testing

In this category, five papers were included. Montgomery et al.^24^ focused on providing an empirical basis to understand the mechanisms through which couple-focused HIV prevention works.^24^ They conducted a comparative qualitative study using in-depth interviews in Uganda and Zambia. Thirty-three interviews were conducted in total: 10 with couples and 23 with staff members at the research sites. The Ugandan site recruited serodiscordant couples and the Zambian site recruited women alone.^24^ Spouses’ transformation of motivation was found to be stronger where couples were recruited as a unit. Coping mechanisms were observed to differ in the two sites; among serodiscordant couples in Uganda, communal coping is evidenced through joint support for adherence and retention. In contrast, coping at the Zambian site was predominantly left to the individual woman and occurred against the backdrop of mutual mistrust and male disenfranchisement.

Allen et al. recruited and trained a broad range of influence network agents (INAs) to promote CVCT through invitations and one-on-one contacts.^17^ Predictors of successful promotion of CVCT were identified using a multi-level hierarchical analysis. In 4 months, 9.900 invitations were distributed by 61 INAs, with 1.411 couples requesting CVCT. INAs in Rwanda distributed fewer invitations but had higher response rates (26.9% vs. 9.6%), than INAs in Zambia. The context of the invitation event, including a discreet location, and the delivery of the invitation to both partners or to someone known to the INA were stronger predictors of success than INA or couple-level characteristics.

In Rakai, Uganda, Kairania et al. evaluated the efficacy of a stepwise strategy to promote disclosure of HIV-seropositive results among HIV-discordant couples.^25^ Two hundred and ninety-three HIV serodiscordant couples were identified through retrospective linkage of married or cohabiting consenting adults individually enrolled into a cohort study and into two randomised trials of male circumcision in Rakai. HIV-serodiscordant couples and a random sample of HIV-positive concordant and HIV-negative concordant couples were invited to sensitisation meetings to discuss the benefits of disclosure and couple counselling. HIV-positive partners were subsequently contacted to encourage disclosure. If the index positive partner agreed, counsellor facilitated the disclosure of HIV results and provided ongoing support. The rates of disclosure were 81.3% in male HIV-positive and 80.2% in female HIV-positive discordant couples.

Jones et al. described the implementation of HIV prevention in a resource-limited setting.^26^ A partner project was implemented within HIV counselling and testing programmes in six urban community health clinics (CHC) in Lusaka, Zambia. One hundred and ninety-seven HIV seroconcordant and discordant couples were sequentially enrolled to the control group or to receive the intervention from partner research or CHC staff members. Couple members completed assessments on condom use, alcohol use and intimate partner violence (IPV) at baseline, 6 months and 12 months follow-up. Sexual barrier use outcomes achieved by the CHC staff were comparable to or better than those achieved by the partner project research staff, and both were superior to the control group. A reduction in IPV was observed for the entire sample.

Allen et al. described the sexual behaviour of 963 cohabiting heterosexual couples following HIV counselling and testing. Biological markers were used to assess the validity of self-report.^16^ Couples were recruited from a same-day VCT centre in Lusaka, Zambia. Sexual exposures with and without condom use recorded at three monthly intervals. Sperm detected on vaginal smears, pregnancy, syphilis and *Trichomonas vaginalis* were assessed. Less than 3% of couples reported current condom use prior to VCT. In the year after VCT, more than 80% of reported acts of intercourse in discordant couples included condom use. Reporting 100% condom use was associated with 39% – 70% reductions in biological markers. Under-reporting was common: 50% of sperm and 32% of pregnancies and HIV transmissions were detected when couples had reported always using condoms.

#### HIV risk reduction

In a prospective cohort study of 53 HIV serodiscordant couples, Allen et al. assessed the impact of an education and CVCT intervention on condom use and HIV seroconversion by using educational videos and facilitated discussion groups.^27^ The rate of condom use increased from 4% to 57% over a 1-year follow-up period.

### Summary of the study findings

There was considerable heterogeneity among studies in terms of trial characteristics, participants included and intervention content, behavioural and biological outcomes. The studies included in this review described outcomes on unprotected sex, reduced HIV transmission, treatment adherence, efficacy of a behavioural intervention and the general acceptability and effectiveness of such intervention programmes. These programmes have reported positive results, but based on the sparse literature on this issue, it appears that they were not scaled up or taken up by other countries.

## Discussion

A combination of interventions including behavioural and biomedical interventions is urgently needed to increase knowledge of HIV status as well as reduce the risk of HIV transmission within married and cohabiting couples.^29^ A review of literature of an NIH-supported study in the United States revealed that behavioural and biomedical interventions for serodiscordant couples should include the following: promotion of couples counselling, testing and disclosure condom promotion, alcohol risk reduction, provision of ARVs, post-exposure prophylaxis, medical male circumcision and prompt treatment of sexually transmitted infections.^29^ Furthermore, a meta-analysis of previous interventions for serodiscordant couples found that most prevention interventions were less effective for African-American communities, highlighting the need for culturally congruent approaches.^12^

In recent times, couple-focused research has gained momentum, with empirical reviews and theoretical frameworks proliferating.^12,24,26^ In a systematic review of studies testing whether couples-based behavioural interventions reduce HIV transmission and risk behaviour, Burton et al. found that the results across studies consistently indicated that couples-focused programmes reduced unprotected sexual intercourse and increased condom use.^10^ Not only have couple-level interventions been demonstrated to be effective, there is also evidence that they are feasible, acceptable and cost-effective.^30^ Although the challenges to recruiting couple cohorts should not be underestimated, various studies have reported ways to overcome these and the importance of doing so.^17^

This review included eight studies of psychosocial and behavioural interventions which included HIV serodiscordant couples. All the studies were conducted in African countries, that is, Uganda, Rwanda, Kenya, Tanzania and Zambia. Each of these studies indicates that participation in couples-focused educational or counselling programmes was associated with reduced HIV transmission, reduced unprotected sex or some behaviour modification, for example, reduced IPV. A closer examination of the main findings across these studies indicates promising effects for HIV prevention programmes that address couples and dyadic relationship issues.^10^

The limitations of the review include a search that only looked at studies that were conducted in Africa. Although limiting our ability to generalise our findings beyond Africa, our primary aim was to focus on this continent which carried the highest burden of HIV infections. The studies that we reviewed varied in the methods that were employed, inclusion and exclusion criteria, sample size and intervention content. Other studies included counsellors or facilitator reports. Our findings may have been influenced by the small sample size, and any generalisations based on these results may be inappropriate.

The key weakness which we uncovered relates to the specific content of the interventions that were reported. For example, as highlighted in the introduction, HIV serodiscordant couples face other challenges such as lack of disclosure, poor communication patterns and the desire for fertility and reproduction.^31^ Ensuring culture congruency was also found to be lacking in most of these interventions; however, previous successful interventions have highlighted the importance of culturally congruent interventions.^12^ A study by McGrath et al. applied the same group intervention to 43 couples: 15 in India, 14 in Thailand and 14 in Uganda but the modules were tailored for local appropriateness to demonstrate the feasibility of implementing group interventions across cultural contexts.^32^

HIV is part of the relationship, and exploring communication among HIV serodiscordant couples is essential for the dyad.^31^ Disclosure of one’s HIV status to a sexual partner has increasingly gained prominence as a challenge in the management of the HIV and AIDS pandemic.^25^ Individuals do not know how to disclose their HIV-positive status and were concerned about testing that did not include treatment and/or follow-up support, as well as the ensuing psychological turmoil in the event of testing HIV positive. Confusion often arises when serodiscordance is identified at post-test counselling and disclosure when it is of vital importance for the couple to have good communication skills. Kairania et al. found that a facilitated couple counselling approach to disclosure among HIV-discordant couples resulted in high rates of disclosure.

Pregnancy in discordant couples is common,^33^ and the desire for more children may partially explain persistent HIV incidence among HIV-discordant couples in Africa and elsewhere. HIV-discordant heterosexual couples are faced with the dual challenge of preventing sexual HIV transmission and unplanned pregnancies with the attendant risk of perinatal HIV transmission.

The unique needs of serodiscordant couples are largely missing from many current family planning efforts which focus on the prevention of pregnancies in the absence of the reduction of the risk of HIV and other sexually transmitted infections. Conversely, HIV testing and counselling programmes focus exclusively on condom use without discussion of more effective contraceptive methods. The challenge is to provide discordant couples with family planning choices that effectively manage both their risk of HIV transmission and their fertility goals.

The review process originally yielded a few papers on fertility interventions for HIV-discordant couples, but following the strict application of inclusion and exclusion criteria, those papers were excluded from the review. Cook et al., in Lusaka Zambia, examined the determinants of fertility desires among HIV seroconcordant and discordant couples. Consistent with previous studies identifying a high proportion of HIV-seropositive individuals desiring and seeking pregnancy, nearly half of the participants expressed the desire for children^34^, and the desire for children was often shared among couple members and the strongest predictor of participants’ desire for children was having a partner who wanted children. The inclusion of both members of the couple in efforts to promote safer conception practices among HIV seroconcordant and discordant couples was found to be highly effecting in achieving safe conception.^34^

Finally, despite our systematic and comprehensive attempt to search the literature, this review might not have identified all the relevant studies such as unpublished reports and papers produced in other languages. However, this article is probably the first systematic review of couples-focused psychosocial and behavioural interventions for HIV-discordant couples in this continent. In addition to this, a proper meta-analysis of the effects and outcomes of each intervention was not possible because of study heterogeneity.

## Conclusion

In order to reach the ambitious UNAIDS target to eradicate AIDS by 2030, Africa urgently needs effective HIV prevention interventions that can be disseminated for use in clinical and community settings, with some special emphasis on mixed status couples who continue to drive the HIV epidemic. We need to acknowledge that no single intervention can turn around the current HIV tide in serodiscordant married or cohabiting couples, but any form of psychosocial support of HIV serodiscordant couples which includes messages about the meaning, mechanisms, challenges and implications of serodiscordancy should be supported. Culturally appropriate HIV disclosure and safer sex messages are also needed to improve these partnerships. This review provides preliminary evidence for the effectiveness and feasibility of couple-focused psychosocial and behavioural interventions to reduce HIV transmission.
